# Fatty acid content in epididymal fluid and spermatozoa during sperm maturation in dogs

**DOI:** 10.1186/s40104-017-0148-6

**Published:** 2017-02-08

**Authors:** Daniel S. Ramos Angrimani, Marcilio Nichi, João Diego A. Losano, Cristina F. Lucio, Gisele A. Lima Veiga, Márcia V. M. Junqueira Franco, Camila I. Vannucchi

**Affiliations:** 1 Department of Animal Reproduction, School of Veterinary Medicine and Animal Science, University of São Paulo, Rua Prof. Orlando Marques de Paiva, 87, São Paulo, 05508-270 Brazil; 2 Department of Internal Medicine, Division of Nutrition, University of São Paulo, Av. Bandeirantes, 3900 - 14040-040, Ribeirão Preto, Brazil

**Keywords:** Dogs, Epididymis, Fatty acids, Sperm maturation

## Abstract

**Background:**

During sperm maturation, there is a reorganization of fatty acids from plasmatic membrane of the spermatozoa, which allows higher membrane integrity and acquisition of sperm motility. However, the fatty acid profile during sperm maturation remains unclear in dogs. Thus, the aim of this study was to identify the fatty acids from the epididymal spermatozoa and plasma during the sperm maturation, and observed changes in the motility and plasmatic membrane parameters. Twenty one adult dogs were used, subsequently to bilateral orchiectomy and epididymal storage, sperm samples were collected from the different segments of the epididymis. Samples were evaluated for conventional microscopy, computer-assisted motility analysis, sperm plasma membrane permeability and the fatty acid analysis (lipids were extracted, transmethylated and analyzed by chromatography).

**Results:**

Caput and corpus sperm showed lower values for the motility variables evaluated and plasmatic membrane integrity, indicating different levels of the fatty acids organization. Saturated, monounsaturated and polyunsaturated fatty acids were in higher concentrations in the spermatozoa from epididymis cauda. Highlighting the presence of caprylic, stearic and docosahexaenoic acids.

**Conclusions:**

These findings demonstrate the influence of the fatty acid profile during sperm maturation, assigning physical and chemical changes in sperm cells, essential for fertilization.

## Background

Sperm maturation occurs along the transit through the epididymal regions, which allows for functional and morphological changes in the spermatozoa [1]. After these modifications, the spermatozoa stabilize its plasma membrane through structural changes in lipid profile and, ultimately, acquire progressive motility [2, 3]. In addition, the remodeling events of the sperm plasma membrane are important to provide cellular integrity and fertilization ability [4, 5]. Molecular and biochemical organization are essential in order to prevent sperm damage during storage and transport through both male and female reproductive tract after ejaculation [5].

Sperm plasmatic membrane is composed by a bilayer of phospholipids, with the saturated or unsaturated fatty acids being the functional units [5]. The origin of the plasma membrane lipid content derives from the spermatogonial cell, however, it can be modified during spermatogenesis and sperm maturation [6]. While passing through the first epididymal region (caput), sperm plasmatic membrane is flexible, facilitating lipid remodeling [7]. However, across the other epidydimal segments (corpus and cauda), sperm becomes more stable and able to withstand the damages of the storage area situated at the cauda epididymides [3]. The lipid organization of the spermatozoa during epididymal maturation is mediated by protein and lipid constituents of the epididymal fluid [5, 8, 9]. Hence, sperm plasma membrane changes in accordance with the environment in which sperm is located. Only at the final stage of epididymal maturation, the plasmatic membrane reaches its final composition, which consists, generally, in 70% phospholipids, 25% neutral lipids (mainly cholesterol) and 5% glycolipids [6, 10]. Among the polyunsaturated fatty acids, the docosahexaenoic acid (DHA) represents 50% of the total sperm content [11].

In mammals, the fatty acids composition of the sperm plasmatic membrane is related to physicochemical properties and, consequently, interferes with the functional competence of the sperm cells. DHA is essential for the fluidity of the plasma membrane, and thus for the acquisition of sperm motility and acrosome reaction [12]. Moreover, DHA is related to the physiological occurrence of oxidative stress in sperm cells [13]. The sperm membrane is rich in polyunsaturated fatty acids (PUFAs), which sensitize to the attack of reactive oxygen species. Such lipid peroxidation is related physiologically to several steps of the fertilization process such as sperm hyperactivation, capacitation, acrosome reaction and fertilization [13]. Simultaneously with the aforementioned changes in sperm lipid membrane, sperm motility parameters gradually change along the epididymal transit, from immobility or vibration to progressive movements [2, 14, 15]. During spermiation to epididymal caput, spermatozoa are immobile. On the other hand, progressive motility, speed and linearity is acquired in the epididymal corpus [16]. However, sperm ability to acquire motility depends on changes in plasma membrane composition [17]. Thus, specific studies are required in order to increase the physiological knowledge regarding sperm maturation and to improve the reproductive biotechnologies involved with semen manipulation [18].

Despite the importance of sperm lipid profile, the exact composition of the fatty acids that structure sperm plasma membrane has not yet been explored for epididymal samples in dogs, only in ejaculated semen [19] and seminal plasma [20]. Moreover, the exact mechanism that structures sperm plasma membrane should be investigated in dogs. To our knowledge, the specific modifications in dog sperm and the exact composition of the epididymal fluid have not yet been fully established or elucidated. As the ideal experimental model for humans, the studies concerning sperm maturation in dogs can enable the development of new biotechnologies, such as male contraception, in vitro sperm maturation and treatments associated with sperm maturation [21].

Hence, the aim of this study was to identify the fatty acid profile of epididymal spermatozoa and epididymal fluid during sperm maturation along the epididymides, and also, characterize changes in sperm motility parameters and plasmatic membrane.

## Methods

The present study was approved by the Bioethics Committee of the School of Veterinary Medicine and Animal Science – University of São Paulo (protocol number: 2277/2011). Unless otherwise stated, all chemicals and reagents were purchased from Sigma–Aldrich (St. Louis, MO, USA).

Twenty one mature dogs (aged from 1 to 6 yr) of distinct breeds and body weights were used (Table 1). At macroscopical exam, testis and epididymides were considered normal. All animals were tested against brucellosis, using the *Antigen Rapid Canine Brucella Ab Ttest* (Bioeasy, Minas Gerais, Brazil), detecting the anti-*Brucella canis* IgG antibodies.

**Table 1:** Age, weight and breed of the dogs from which testis and epididymides were harvested (n = 21)

	Breed	Weight, kg	Age, yr
Dog 1	Mixed Breed	19.6	1
Dog 2	Labrador Retriever	26.6	3
Dog 3	Mixed Breed	6.4	1
Dog 4	Mixed Breed	7.8	5
Dog 5	Poodle	12.8	5
Dog 6	Mixed Breed	18	3
Dog 7	Mixed Breed	12.6	2
Dog 8	Mixed Breed	7.8	1
Dog 9	Mixed Breed	14.6	2
Dog 10	Mixed Breed	10.5	2
Dog 11	Mixed Breed	12.8	2
Dog 12	Mixed Breed	17.9	1
Dog 13	Mixed Breed	48	2
Dog 14	Labrador Retriever	19.9	6
Dog 15	Mixed Breed	40	3
Dog 16	German Shepherd	11.9	2
Dog 17	Mixed Breed	14.8	2
Dog 18	Shetland Sheepdog	7.6	6
Dog 19	Schnauzer	7.5	6
Dog 20	Mixed Breed	45	3
Dog 21	Mixed Breed	13.7	1

After the bilateral orchiectomy, testicles–epididymis were immediately stored at 5 °C and samples were transported to the laboratory. Epididymal storage has been proved safe at 4 °C for no more than 24 h [22]. Therefore, after 18–24 h, we collected the epididymal sperm by individual incisions in the caput, corpus and cauda epididymides with a scalpel blade. Dissection was performed carefully to avoid sectioning of blood vessels. The flowing epididymal fluid and sperm was collected with an automatic pipette, and the total volume of the epididymal sample (fluid + sperm) was resuspended in 300 μL of PBS medium (phosphate buffered saline), according to [23]. The dilution rate varied according to the epididymal size, similar to a previously described protocol [24]. To calculate the epididymal sample (fluid + sperm) dilution factor, tubes containing solely the PBS medium were weighted before (tube + PBS) and after the addition of the epididymal sample (tube + PBS + epididymal sample). Due to the small volume recovered, the samples from the left and right epididymides of the same animal were pooled.

Although the ejaculate sperm analysis has not been performed, the epididymal sperm were within adequate parameters for dogs, according to [25] and [23] assuring that testicular and epidydimal abnormalities were ruled out.

### Immediate sperm evaluation

Samples from the epididymal segments (i.e. caput, corpus and cauda) were immediately evaluated for sperm motility and vigor by conventional microscopy (Nikon, Eclipse E200, Japan) at 1,000× magnification and for computer-assisted sperm analysis (CASA; HTM-IVOS Ultimate 12.3; Hamilton Thorne Biosciences, Beverly, MA, USA), according to a previously described protocol [26]. Briefly, 10 μL of each sample was deposited on microscope slides previously warmed at 37 °C and covered by coverslips. Eight fields of view were randomly selected and the following variables were assessed: VAP (average pathway velocity, μm/s), VSL (strait-line velocity, μm/s), VCL (curvilinear velocity, μm/s), ALH (amplitude of lateral head displacement, μm), BCF (beat cross frequency, Hz), STR (straightness -VSL/VAP, %), LIN (Linearity - VSL/VCL,%); MOT (motility, %), PROG (progressive motility, %). Sperm were also divided into four groups based on velocity: rapid (RAP, VAP > 50 μm/s, %), medium (MED, 30 μm/s < VAP < 50 μm/s, %), slow (SLOW, VAP < 30 μm/s or VSL < 15 μm/s, %) and non-moving spermatozoa (STATIC, %).

Samples were also evaluated for plasma membrane permeability using the eosin/nigrosin stain [27]. In brief, 5 μL of semen and 5 μL of the previously prepared stain were placed in a pre-warmed (37 °C) glass slide. The sperm smear was evaluated under light microscopy (Nikon, Eclipse E200, Japan) at 1,000× magnification. We considered intact sperm (membrane integrity) as sperm with no stain; and damaged sperm (membrane lesion) as pink colored cells. Results were analyzed by counting 200 cells and expressed as percentage of stained sperm (%).

### Semen processing for fatty acid analysis

Immediately after semen analysis, samples were centrifuged at 800 ×g for 10 min to separate the spermatozoa from the epididymal fluid. The volume of each sample contained an equivalent concentration of 1.5 million spermatozoa, by pooling the sample of four dogs, totalizing five pools of each segment of the epididymides (caput, corpus and cauda), according to previous works [28, 29]. For the epididymal fluid analysis we used a fixed volume of 60 μL. Samples were stored at −20 °C until analysis.

For the fatty acids extraction, we utilized the transesterification method described by Lepage and Roy [30]. Samples were transferred to a glass cuvette, to which 10 μL of sodium chloride and triphenylphosphate (10 mg/mL; as internal standard) was added. Subsequently, 1 mL of the solution was combined to methanol:acetyl chloride, in a 100:5 proportion (3 mL:150 μL), and the cuvette was maintained at 100 °C for 60 min for fatty acids transmethylation [31].

After incubation, the cuvettes were maintained at room temperature for the addition of 1 mL of hexane, which allowed the solubilization of fatty acids, enabling the passage through gas chromatography. Samples were vortexed for 60 s and then centrifuged (640 ×g for 5 min). The supernatant was transferred to a glass jar and dried by N_2_ vapor. Sequentially, the sample was suspended in 50 μL of hexane, vortexed for 60 s and 1 μL of this solution was injected into the gas chromatograph (GC-17A®, Shimadzu, Kyoto, Japan).

The fatty acids were classified in accordance with the retention time of a pre-established curve, then sorted into saturated fatty acids (butyric, caproic, caprylic, capric, undecanoic, lauric, tridecanoic, myristic, pentadecanoic, palmitic, heptadecanoic, stearic, arachidic, heneicosanoic, behenic, tricosanoic and lignoceric), monounsaturated fatty acids (myristoleic, pentadecenoic, palmitoleic, elaidic, oleic, eicosenoic, erucic and nervonic) and polyunsaturated fatty acids (linoleic, linolelaidic, gamma-linolenic, alpha-linolenic, eicosadienoic, arachidonic, eicosatrienoic, eicosapentaenoic acid and docosahexaenoic).

### Stastical analysis

All data were analyzed using the SAS for Windows (SAS Institute Inc., Cary, NC, USA). The effect of sperm origin (epididymal spermatozoa from caput, corpus and cauda) and the matrix used (epididymal fluid vs. sperm) on fatty acids content was determined using parametric (Student’s *t* test for two treatments or LSD test for more than two treatments) and nonparametric (Wilcoxon) tests, according to the residue normality (Gaussian distribution) and variance homogeneity of each variable. The different fatty acids were also divided, grouped and analyzed according to the number of unsaturations (i.e. saturated, monounsaturated and polyunsaturated).

A probability value of *P* < 0.05 was considered statistically significant. The results are reported as untransformed means ± SEM. Pearson’s correlation was used to calculate the relationship between the variables studied in each variable group. In order to perform the correlation analysis between the concentration of fatty acids and the sperm analysis, data (i.e. sperm motility and plasma membrane integrity) were merged according to the pool of samples adopted for the fatty acids analysis.

## Results

Samples from the epididymal cauda presented a higher sperm motility and vigor in comparison to the corpus and caput segments, respectively (Table 2). The same pattern was observed for the percentage of sperm with plasmatic membrane integrity, i.e., cauda sperm had greater integrity compared to the corpus and caput (Table 2). The Computer Assisted Semen Analysis (CASA) showed significant and progressive increase in sperm motility, progressive motility, rapid speed, average path velocity (VAP), straight line velocity (VSL), curvilinear velocity (VCL), straightness (STR) and linearity (LIN), while passaging through epididymal segments (caput, corpus and cauda) (Table 2). The sperm variables related to medium speed, amplitude of lateral head displacement (ALH) and beat cross frequency (BCF) were not different between the corpus and cauda groups, while caput sperm showed lower results (Table 2). The index of spermatozoa with progressive motility in the caput did not differ from the corpus samples, but was lower than the cauda of the epididymides (Table 2). However, the percentage of static spermatozoa from the epididymis caput was greater than corpus and cauda sperm (Table 2).

**Table 2:** Effect of sperm origin (epididymal cauda, corpus and caput) on subjective sperm motility, vigor, plasma membrane integrity and computer assisted sperm analysis (CASA) in dogs

	Caput	Corpus	Cauda
Sperm motility, %	0.0 ± 0.0^c^	27.7.4 ± 3.0^b^	69.7 ± 4.0^a^
Sperm vigor (1–5)	0.0 ± 0.0^c^	2.0 ± 0.1^b^	2.6 ± 0.1^a^
Intact plasma membrane, %	40.1 ± 4.7^c^	74.5 ± 2.3^b^	92.6 ± 1.1^a^
% of Motile	0.0 ± 0.0^c^	34.5 ± 3.5^b^	71.0 ± 4.4^a^
% of Progressive	0.0 ± 0.0^b^	5.5 ± 0.9^b^	27.8 ± 2.9^a^
% of Rapid	0.0 ± 0.0^c^	18.2 ± 2.4^b^	54.2 ± 4.5^a^
% of Medium	0.0 ± 0.0^b^	16.3 ± 1.7^a^	16.8 ± 2.6^a^
% of Slow	6.0 ± 2.5	6.0 ± 2.4	10.5 ± 1.4
% of Static, %	94.0 ± 2.5^a^	54.8 ± 4.4^b^	23.1 ± 3.6^c^
VAP, μm/s	0.0 ± 0.0^c^	66.3 ± 5.5^b^	94.9 ± 5.6^a^
VSL, μm/s	0.0 ± 0.0^c^	37.8 ± 3.7^b^	67.2 ± 4.6^a^
VCL, μm/s	0.0 ± 0.0^c^	142.5 ± 10.3^b^	178.5 ± 10.0^a^
ALH, μm/s	0.0 ± 0.0^b^	7.3 ± 0.6^a^	7.8 ± 0.3^a^
BCF, μm/s	0.0 ± 0.0^b^	32.0 ± 3.1^a^	28.3 ± 2.2^a^
Straightness, STR - %	0.0 ± 0.0^c^	48.9 ± 3.5^b^	67.4 ± 1.4^a^
Linearity, LIN - %	0.0 ± 0.0^c^	26.3 ± 2.4^b^	38.9 ± 1.5^a^

Different superscripts in the same line indicate significant differences (*P* < 0.05)

In the corpus segment, there was a positive correlation between sperm straightness and the percentage of sperm with intact plasmatic membrane (r = 0.49; *P* = 0.03). In the epididymides cauda, a positive correlation was observed between the percentage of sperm with intact membrane and subjective motility (r = 0.80; *P* < 0.0001), CASA motility (r = 0.63; *P* = 0.003) and progressive motility (r = 0.46; *P* = 0.04).

**Fig. 1:**
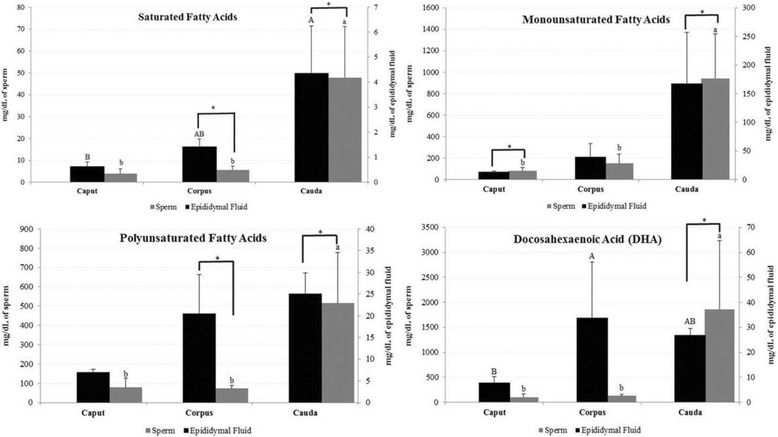
Mean and standard error ($\overline{\mathrm{X}}$ ± SE) of saturated (×10^3^ mg/dL), monounsaturated (mg/dL), polyunsaturated fatty acids (mg/dL) and docosahexaenoic acid (DHA; mg/dL) in sperm and epididymal fluid according to the epididymal segment (caput, corpus and cauda) in dogs. ^a,b^values with different superscript letters differ significantly between segments for epididymal sperm (*P* < 0.05). ^A,B^values with different superscript letters differ significantly between segments for epididymal fluid (*P* < 0.05). *Indicates statistical difference between epididymal fluid and sperm (*P* < 0.05)

Regarding the identification of the saturated fatty acids, we observed higher content in the epididymal fluid from epididymal cauda in comparison to the caput, but not different from the epididymal corpus (Fig. 1). On the other hand, sperm samples from the cauda had higher concentration of saturated fatty acids compared to epididymal corpus and caput (Fig. 1). Regarding the matrix origin, sperm had higher concentration of saturated fatty acids compared to the epididymal fluid in the cauda (*P* = 0.0005) and corpus segments (*P* = 0.004) (Fig. 1).

No difference was observed between caput, corpus and cauda for monounsaturated and polyunsaturated fatty acid concentrations in the epididymal fluid (Fig. 1). However, sperm from the epididymal cauda presented higher monounsaturated and polyunsaturated fatty acid content compared with the corpus and caput of the epididymis (Fig. 1). Moreover, regarding the matrix origin, sperm had higher concentration of monounsaturated and polyunsaturated fatty acids compared to the epididymal fluid in the caput (*P* = 0.05) and cauda (*P* = 0.05) segments, and in the corpus (*P* = 0.01) and cauda epididymides (*P* = 0.04), respectively (Fig. 1). For the concentration of docosahexaenoic acid (DHA; Fig. 1), we observed statistical difference between the epididymal fluid and sperm in the cauda of epididymis (*P* = 0.005). Moreover, corpus epididymal fluid had greater DHA concentration compared only to the caput, while cauda sperm present the highest content compared to the other epididymal segments (Fig. 1). In the fluid and sperm of the epididymal caput, corpus and cauda we observed higher concentration of satured fatty acid in comparison to monounsaturated and polyunsaturated (Table 3).

**Table 3:** Fatty acid concentration (%) of saturated, monounsaturated and polyunsaturated in sperm and epididymal fluid of each epididymal segment (caput, corpus and cauda) in dogs

	Cauda		Corpus		Caput	
	Edidymal Fluid	Sperm	Edidymal Fluid	Sperm	Edidymal Fluid	Sperm
Satured	95.76%	97.04%	95.99%	96.18%	96.92%	96.05%
Monounsatured	3.68%	1.91%	2.63%	2.57%	2%	2.01%
Polyunsaturated	0.54%	1.04%	1.37%	1.24%	1.07%	1.92%

In the epididymal fluid, the following fatty acids were identified: caprylic, capric, heptadecanoic and nervonic, without statistical difference between caput, corpus and cauda (Table 4). However, the concentration of stearic and pentadecenoic fatty acids were higher in the epididymal cauda in comparison to the corpus and caput, and the linoleic fatty acid had lower concentration in the epididymal caput compared with the corpus and cauda, which did not differ significantly (Table 4).

**Table 4:** Mean and standard error ($\overline{\mathrm{X}}$ ± SE) of the epididymal fluid fatty acids (mg/dL) according to the epididymal segment (caput, corpus and cauda) in dogs

	Caput	Corpus	Cauda
Caprylic	31,35.84 ± 1,640.87	3,467.34 ± 917.79	25,493.90 ± 20,432.01
Capric	25.15 ± 19.05	11.25 ± 1.25	25.36 ± 9.78
Heptadecanoic	8.36 ± 1.36	9.47 ± 0.63	23.15 ± 10.78
Nervonic	15.11 ± 3.31	16.72 ± 1.49	41.52 ± 10.56
Stearic	33.10 ± 5.64^a^	68.85 ± 11.35^a^	171.19 ± 26.22^b^
Pentadecenoic	17.06 ± 4.33^a^	24.18 ± 3.45^a^	129.21 ± 20.21^b^
Linoleic	4.68 ± 0.75^a^	10.49 ± 2.50^b^	11.03 ± 1.17^b^

Different superscripts in the same line indicate significant differences (*P* < 0.05)

Regarding the fatty acids content in the epididymal sperm (Table 5), caproic and capric fatty acids were present in all epididymal segments, although without statistical difference (Table 5). Concentrations of caprylic, pentadecenoic and stearic were lower in the caput and corpus in comparison to the epididymal cauda (Table 5). The concentration of linoleic fatty acid was higher in cauda epididymides compared to epididymal corpus, without statistical difference with the epididymal caput.

**Table 5:** Mean and standard error ($\overline{\mathrm{X}}$ ± SE) of the sperm fatty acids (mg/dL) according to the epididymal segment (caput, corpus and cauda) in dogs

	Caput	Corpus	Cauda
Caproic	21.49 ± 13.06	133.54 ± 83.10	1,486.92 ± 1,040.43
Capric	10.34 ± 1.11	109.02 ± 68.63	261.38 ± 94.15
Caprylic	13,915.63 ± 9,278.72^a^	15,273.23 ± 5,796.17^a^	166,899.87 ± 92,944.15^b^
Stearic	137.76 ± 43.45^a^	159.02 ± 12.25^a^	1,385.99 ± 517.63^b^
Pentadecenoic	92.66 ± 18.70^a^	75.73 ± 11.47^a^	939.84 ± 415.22^b^
Linoleic	72.34 ± 61.94^AB^	40.06 ± 5.71^a^	222.23 ± 77.36^b^

Different superscripts in the same line indicate significant differences (*P* < 0.05)

## Discussion

In the present study, we evaluated sperm motility, plasmatic membrane integrity and fatty acid profile of spermatozoa harvested from the caput, corpus and cauda of the epididymides in dogs. The lipid analysis was performed in the epididymal sperm and fluid as well.

We observed higher concentration of saturated, monounsaturated and polyunsaturated fatty acids in the sperm harvested from the epididymal cauda in comparison with the corpus and caput. This result can be attributed to the necessary acquisition of lipids during the epididymal maturation, which will further act during sperm capacitation and oocyte fertilization in the female tract [32]. Moreover, we observed higher concentration of specific fatty acids. For saturated fatty acids, the caprylic and stearic fatty acids were more prominent. The former fatty acid has antimicrobial and protective functions in the sperm membrane [33], while stearic fatty acid is proved to regulate bovine sperm function, providing energy metabolism and sperm motility [34]. Therefore, increased concentrations of caprylic fatty acid in the spermatozoa of the epididymal cauda may be responsible for the greater integrity of the plasmatic membrane, also observed through our sperm membrane integrity stain analysis.

There was a prevalence of satured fatty acids in all epididymal segments in both spermatozoa and fluid, similar to results obtained by Martinez-Soto, Landeras [11] in men. It is possible to infer that the high percentage of satured fatty acids can minimize the eventual sperm damage during the long term storage in the epididymis, since polyunsaturated fatty acids are more easily oxidized by reactive oxygen species (ROS) [11, 35]. However, this result must be further confirmed by future studies on oxidative stress challenge of stored epididymal sperm.

In the present experiment, the pentadecenoic fatty acid was the predominant monounsaturated fatty acid in the spermatozoa, whereas the docosahexaenoic (DHA), the main polyunsaturated fatty acid. Among the polyunsaturated fatty acids, DHA represents 50% of the total content of human spermatozoa [11]. In boars, DHA content increases during sperm epididymal maturation [36]. Unlike other cells, mature sperm presents a high quantity of polyunsaturated fatty acids at plasma membrane level [37], providing sperm integrity [38] and progressive motility [39], in addition to being considered necessary for the fluidity of the plasma membrane. Therefore, it is possible to affirm that an increased sperm DHA concentration is essential for the final steps of epididymal maturation in dogs, because it is directly involved in events required for fertilization (i.e. motility and plasmatic membrane integrity). Additionally, there was a lack of flagellum movement in the caput, onset of motility in the epididymal corpus and higher percentage of spermatozoa with progressive flagellar movement in the epididymal cauda. Sperm maturation requires changes in the lipid structure of sperm plasma membrane, aiming an efficient transduction of ATP from mitochondria, which is essential for sperm motility acquisition [40]. In the present work, a positive correlation between sperm straightness and progressive motility and plasmatic membrane integrity in the epididymal corpus and cauda, respectively, were verified. Therefore, in dogs, the observed increase in sperm motility in the epididymal caput and corpus is presumably related to plasma membrane integrity, which can be dependent on the incorporation of polyunsaturated fatty acids to the sperm plasma membrane at the end of epididymal maturation.

Regarding the total fatty acids content (saturated, monounsaturated and polyunsaturated), we observed differences in sperm lipid profile throughout epididymal maturation. Modifications on the primary lipid structure of the sperm plasmatic membrane are related to adhesion of proteins and fatty acids of the epididymal lumen [1, 5, 17]. Thus, modifications during sperm maturation in the epididymides occur according to the protein and lipid profile of the epididymal fluid [41]. In fact, in our experiment, a higher concentration of fatty acids, such as stearic and pentadecenoic fatty acids, was observed in the epididymal cauda fluid, where sperm reaches its highest degree of maturation. Such fatty acids were observed previously in ejaculated semen in dogs [20, 42]. In addition, we observed increase in saturated fatty acids between the epididymal corpus and cauda, but not for DHA concentration. Therefore, it can be inferred that during the sperm transit between corpus and cauda epididymides, DHA from the epididymal lumen was incorporated into sperm cells, thus consuming the DHA concentration of the epididymal fluid and increasing the lipid concentration of the sperm plasmatic membrane. For these reasons, in dogs, fatty acids of the epididymal fluid may act as signaling and regulators of the lipid secretion by the epididymal cells at caput and corpus level, as they are later incorporated into the spermatozoa at the final stages of sperm maturation.

## Conclusions

In conclusion, caput and corpus sperm are immature and have low fertilizing potential, as decreased sperm motility and plasma membrane integrity were verified at such stages of sperm maturation in dogs. However, cauda sperm acquire motility and membrane integrity simultaneously to changes in sperm plasma membrane lipid content through epididymal transit. Moreover, sperm lipid profile derives from incorporation of fatty acids from the epididymal fluid, thus, modulating the biochemical content of the sperm plasma membrane.

Our results indicate that the use of spermatozoa from epididymal cauda can be a viable alternative for reproductive biotechnologies in canids, since cauda sperm shows similarities with ejaculated semen in regards to motility and membrane integrity. Furthermore, this study provides information for future researches on contraception, such as the use of inhibitors of the enzymes that are responsible for fatty acid biosynthesis (e.g., stearoyl-CoA desaturase), promoting lipid disruption of sperm from epididymal cauda. On the other hand, the present data also contribute to gamete manipulation techniques, e.g., in vitro sperm maturation, highlighting the importance of fatty acids supplementation (e.g., DHA, caprylic and stearic fatty acids) for immature canine spermatozoa. Furthermore, there is a need for further studies using a larger number of animals in order to improve our comprehension on the lipid modulation during the epididymal maturation in dogs.
